# The low-cost Shifter microscope stage transforms the speed and robustness of protein crystal harvesting

**DOI:** 10.1107/S2059798320014114

**Published:** 2021-01-01

**Authors:** Nathan David Wright, Patrick Collins, Lizbé Koekemoer, Tobias Krojer, Romain Talon, Elliot Nelson, Mingda Ye, Radosław Nowak, Joseph Newman, Jia Tsing Ng, Nick Mitrovich, Helton Wiggers, Frank von Delft

**Affiliations:** a Structural Genomics Consortium, University of Oxford, ORCRB, Roosevelt Drive, Oxford OX3 7DQ, United Kingdom; b I04-1, Diamond Light Source Ltd, Harwell Science and Innovation Campus, Didcot OX11 0QX, United Kingdom; c Oxford Lab Technologies Ltd, Kemp House, 160 City Road, London EC1V 2N, United Kingdom; d Faculty of Science, University of Johannesburg, Auckland Park, Johannesburg 2006, South Africa

**Keywords:** protein crystal mounting, automation, X-ray crystallography, fragment screening, *X*–*Y* stage, microplates, structural genomics, high-throughput screening, COVID-19, MPro

## Abstract

Despite the tremendous success of X-ray cryo-crystallography in recent decades, the transfer of crystals from the drops in which they are grown to diffractometer sample mounts remains a manual process in almost all laboratories. Here, the Shifter, a motorized, interactive microscope stage that transforms the entire crystal-mounting workflow from a rate-limiting manual activity to a controllable, high-throughput semi-automated process, is described. By combining the visual acuity and fine motor skills of humans with targeted hardware and software automation, it was possible to transform the speed and robustness of crystal mounting. Control software, triggered by the operator, manoeuvres crystallization plates beneath a clear protective cover, allowing the complete removal of film seals and thereby eliminating the tedium of repetitive seal cutting. The software, either upon request or working from an imported list, controls motors to position crystal drops under a hole in the cover for human mounting at a microscope. The software automatically captures experimental annotations for uploading to the user’s data repository, removing the need for manual documentation. The Shifter facilitates mounting rates of 100–240 crystals per hour in a more controlled process than manual mounting, which greatly extends the lifetime of the drops and thus allows a dramatic increase in the number of crystals retrievable from any given drop without loss of X-ray diffraction quality. In 2015, the first in a series of three Shifter devices was deployed as part of the XChem fragment-screening facility at Diamond Light Source, where they have since facilitated the mounting of over 120 000 crystals. The Shifter was engineered to have a simple design, providing a device that could be readily commercialized and widely adopted owing to its low cost. The versatile hardware design allows use beyond fragment screening and protein crystallo­graphy.

## Abstract 2

A motorized *X*–*Y* microscope stage is presented that combines human fine motor control with machine assistance and automated experiment documentation in order to transform productivity in protein crystal harvesting.

## 1. Introduction   

Since the turn of the century, macromolecular crystallography (MX) has undergone a revolution in productivity to become a high-throughput technique, thanks in a large part to machine automation. Nanolitre-scale liquid handlers and robotic microplate imagers (Kuhn *et al.*, 2002 ▸; Stevens, 2000 ▸) are common in many laboratories. Access to bright X-ray sources, high-speed X-ray detectors and cryogenic sample changers that allow complete X-ray data sets to be measured in less than a minute (Bowler *et al.*, 2015 ▸; Grimes *et al.*, 2018 ▸) is also routine. The notable exception to this trend in automation has been the transfer of protein crystals from the crystallization drop to the sample mounts where they are stored for later X-ray diffraction.

Manual mounting is an easy skill to learn but a difficult one to master. Minimizing experimental variability between crystals and maximizing mounting productivity requires simultaneous management of multiple challenges: fine movements and sensory input to manipulative crystals gently, awareness of changing drop conditions, organization of multiple sample plates and thorough data management. Unsurprisingly, manual mounting of crystals presents a source of experimental variability and sample loss, and is a bottleneck in the wider MX workflow.

One strategy to eliminate the mounting bottleneck has been to avoid the need for transfer entirely by developing *in situ* diffraction techniques (Bingel-Erlenmeyer *et al.*, 2011 ▸; Michalska *et al.*, 2015 ▸; Soliman *et al.*, 2011 ▸). Other approaches to *ex situ* screening have tried to design humans out of the mounting process, either via novel harvesting techniques or by reproducing the human mounting technique using advanced robotics (Cipriani *et al.*, 2012 ▸; Deller & Rupp, 2014 ▸; Viola *et al.*, 2007 ▸, 2011 ▸). However, in most laboratories the harvesting step remains the same delicate, labour-intensive and manual process that it was at the advent of cryo-crystallography (Garman & Schneider, 1997 ▸; Juers *et al.*, 2018 ▸). The lack of an accessible solution to the crystal-transfer bottleneck means that the systemic inefficiency in MX remains.

As experimental throughput is increased, manual mounting and experimental documentation become limiting. For instance, in the 11 years from April 2004 to 2015 the 1718 structures released to the Protein Data Bank by the Structural Genomics Consortium (SGC) involved mounting 48 000 crystals by hand. The time taken up by manual mounting and data management at this scale places an artificial limit on the kinds of experiments that can be performed. Crystal fragment screening, which requires hundred to thousands of crystals per target, is one use case that is limited by the absence of a solution to crystal transfer.

No solution to the mounting bottleneck has yet achieved widespread adoption, for a variety of possible reasons: incompatibility with existing practices, high initial cost, engineering support burden or a lack of commercial availability. Perhaps the most fundamental obstacle to the full automation of X-ray crystallography is the technical difficulty that crystal harvesting presents. Locating crystals requires high-resolution imaging in space and in time. Crystals can move by as much as 15 µm s^−1^ owing to fluid dynamical effects (Savino & Monti, 1996 ▸) and will be further disturbed during mounting (Read & Meyer, 2000 ▸). Whilst most crystallography laboratories will have a stereoscopic microscope for manual mounting, stereoscopic digital imaging systems do not seem to offer a sufficient *z*-axis resolution at the necessary frame rate (Kwon *et al.*, 2010 ▸; Pei *et al.*, 2012 ▸; Dean *et al.*, 2017 ▸; Štolc *et al.*, 2014 ▸; Levoy *et al.*, 2006 ▸). Furthermore, the identification of protein crystals from digital images by software has proven to be exceptionally difficult because of significant variations between imaging conditions and crystal appearance; crystals are often very small (∼10–75 µm), colourless, display poor optical contrast with the surrounding droplet (Nollert, 2003 ▸) and can be obscured by other droplet features. A significant body of research has accumulated since the emergence of high-throughput MX on the problem of accurately identifying crystals using automated image analysis (Liu *et al.*, 2008 ▸; Ng *et al.*, 2014 ▸; Bruno *et al.*, 2018 ▸), yet solutions have been only partial.

Fully automated crystal mounting, compatible with existing experimental practice and accessible to the wider community, may be some years off (Deller & Rupp, 2014 ▸). Sensing and manipulating fragile protein crystals requires fine movements and rapid sensory feedback, which are complex and costly engineering challenges to resolve. In view of this, our approach motorizes and automates only that which is easy to engineer, while making it easy for the mounter to contribute what humans are good at. We show that the resultant instrument is effective in greatly speeding up the harvesting process, producing better crystals, and that the solution has the potential to become widely used in crystallography and other fields.

## 2. Design and operation of the Shifter   

### 2.1. Overview of the device and how it is used   

**Figure 1:**
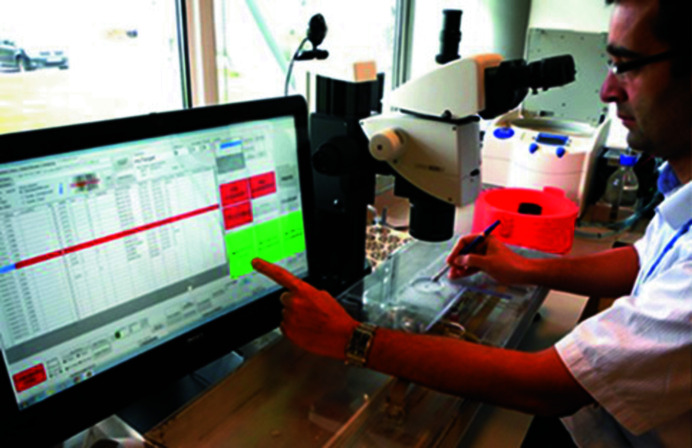
The Shifter at XChem. The operator is mounting through the mounting aperture, whilst operating the GUI with the free hand. The interface manoeuvres samples within the device enclosure, whilst automatically completing experimental annotations and documentation.

The Shifter is a motorized *X*–*Y* stage allowing one or two microplates (ANSI/SLAS 2004) to be loaded via a port in the protective enclosure lid. Immediately before loading, the film seals are completely removed from the microplates. The operator uses a touch-screen PC to move sample droplets to a hole in the lid at the microscope optical axis, where the crystals can be harvested by the scientist using loops in the normal manner. Mounting locations are selected through a graphical user interface (GUI), including from a list populated from a database (Fig. 1 ▸) (von Delft, 2016 ▸; Krojer *et al.*, 2017 ▸).

### 2.2. The device hardware   

**Figure 2:**
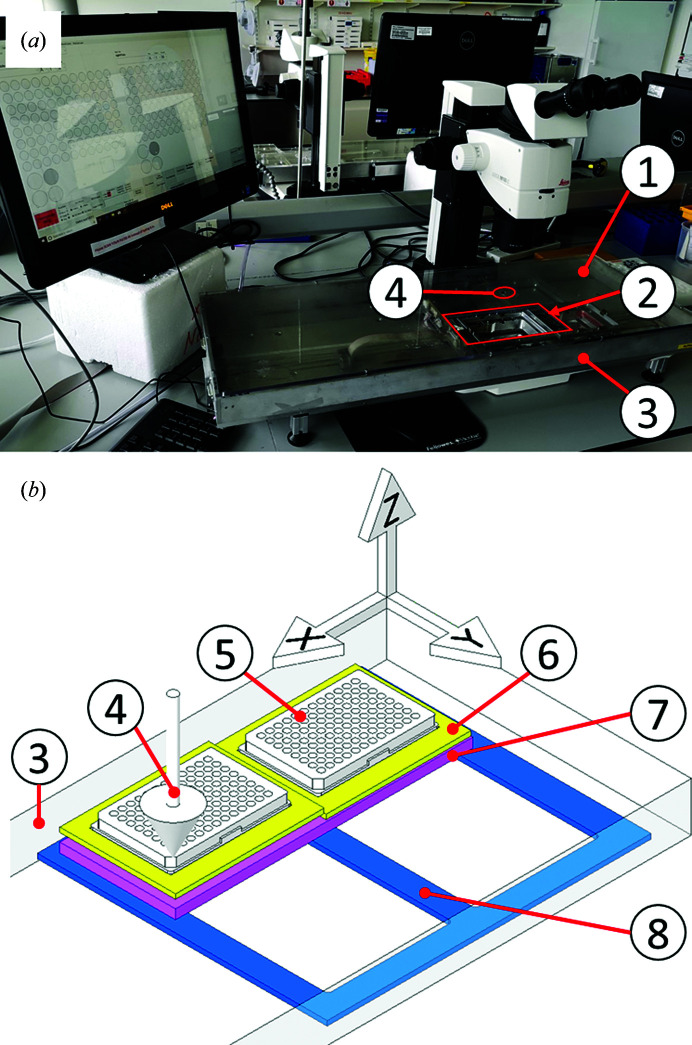
(*a*) The Shifter as installed at a mounting microscope. (1) Clear protective enclosure lid. (2) Port for loading sample plates. (3) Shifter enclosure (∼90 × 30 × 6 cm). (4) Mounting aperture/optical axis. (*b*) A simplified drawing of the main elements of the sample stage within the Shifter enclosure, showing the microplates (5) in their plate carriers (6), which are mounted on the *Y*-axis carriage (7), all of which travels on and with the *X*-­axis carriage (8).

The Shifter enclosure is metal (labelled 3 in Figs. 2 ▸ *a* and 2 ▸ *b*), with a clear plastic lid (labelled 1) that has a large port for loading plates (labelled 2) and a mounting aperture to access the loaded plates that is concentric with the optical axis of the microscope (labelled 4). The Shifter is installed at a mounting microscope (Fig. 2 ▸ *a*). Microplates, loaded with the seals completely removed, are manoeuvred in the *X* (left-to-right) and *Y* (front-to-back) directions by means of stepper motors and toothed belts, with positional feedback from linear encoders (Spectra Symbol, Salt Lake City, Utah, USA). Any part of the left or right microplate can be positioned at the mounting aperture for human mounting, whilst every other part of the microplate remains sealed.

The *X*-axis carriage (labelled 8 in Fig. 2 ▸ *b*) moves in tracks on the enclosure base on low-friction polymer linear slide bearings (igus GmbH, Cologne, Germany). The *Y*-axis carriage (labelled 7) travels in guide tracks on top of the *X*-axis carriage using a similar method of transmission. Two independent plate carriers (labelled 6) hold one microplate (labelled 5) each and move in the *Z* axis. This low-cost stage construction contrasts with typical motorized microscope-stage construction, which often use high-cost, precision-made components that also require tighter tolerances in the manufacture of the assemblies to which they are mounted.

### 2.3. The device supports diverse experimental protocols   

The motor control and encoder feedback are coordinated via a Windows Form Application (.NET Framework) developed using the Microsoft Visual Studio integrated development environment (IDE) and coded in C#. Low-level functions convert real-world microplate and stage dimensions into target encoder values and issue motor moves. Above this motor-control and encoder-feedback layer, any user interface or workflow can be implemented; thus, the Shifter could be adapted and used widely in differing applications. Finally, a prototype graphical user interface (GUI) was developed to allow the high-level driving of stages to specific microplate locations and also to explore how best to support users in one specific workflow.

**Figure 3:**
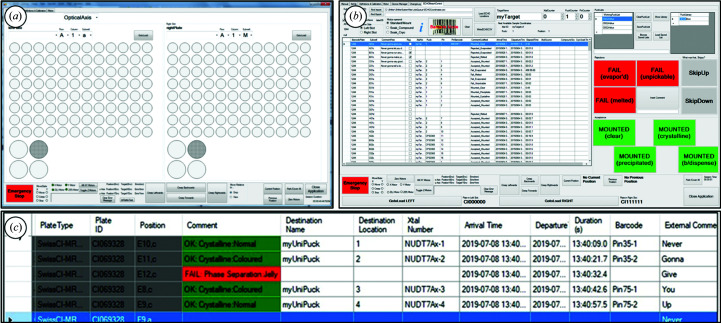
(*a*) An array of GUI buttons allows any subwell to be selected and driven to the mounting aperture. These moves are not annotated. (*b*) Guided crystal-mounting interface; the table contains an imported picklist of the locations to be mounted from. Touch-screen Outcome buttons (shown in red/green) trigger automated experimental annotation and drive the stage to the next location in the list. (*c*) A close-up of the picklist table with columns for tracking data (plate or crystal trial data, pin or mount data) and experimental outcomes, which are populated automatically in real time by the Outcome buttons.

In non-workflow operation, the user can view or mount from droplets by selecting the microplate and subwell via an array of buttons using a computer touch screen (Fig. 3 ▸ *a*). The stage drives the requested location to the mounting aperture. Since this is not a mode specifically intended for experimental work, annotations are not captured.

The implemented use case was the XChem experiment, which entails harvesting crystals from a list of drops in a microplate which have typically been pre-soaked with fragment compounds or solvent or otherwise (Collins *et al.*, 2017 ▸). Locations of interest are known in advance and are therefore imported into a GUI table in the form of a CSV (comma-separated variable) file (Fig. 3 ▸ *b*); this file can contain locations from any number of plates or experiments. When a location in the table is selected by the user or activated by the software, it is moved to the mounting position, allowing the user to mount crystals from this location and record their actions and observations using large touch-screen Outcome buttons. These buttons are configurable and allow the user to use their nonmounting hand to simultaneously progress through the table of crystal locations conveniently while capturing the experimental outcome, while sample-tracking data are automatically generated (Fig. 3 ▸ *c*). Manually entered comments can also be recorded if desired. These data are exported in real-time to a CSV file that can be uploaded into the user’s general workflow tracker, for example a database or a laboratory information-management system (LIMS).

The data thus captured can be used to establish correlations between experiment outcomes and protocols, something that has historically been very hard to achieve. This was the principal data source for the statistical analyses in this study.

To demonstrate the flexibility of the Shifter hardware to support two experimental protocols, further sample-format adaptations were implemented.

#### 2.3.1. Protocol 1: simple guided mounting   

In simple mounting mode, one or two microplates are loaded at a time from the list locations imported into the interface (Fig. 3 ▸ *b*). As users navigate down the work list, the relevant plate and location is physically moved to the mounting aperture. When the loaded plates have been processed, the user is prompted to load the next plates in the series. Annotations are saved as previously described.

#### 2.3.2. Protocol 2: fragment soaking   

This user-tailored workflow facilitates cryoprotectant flash-soaking and harvest–soak–retrieve compound-soaking processes. Here, the user operates a table of crystal source locations and a second table of soak locations. Navigating between the two lists, source-plate and destination-plate locations are presented at the mounting aperture, where crystals are mounted, transferred to the soak condition and later retrieved. Fields in the user work list are automatically populated with tracking data linking unique crystal identities to soak conditions for export as a CSV file.

#### 2.3.3. Hardware adaptations to additional microplate formats   

Large-droplet format hanging-drop and sitting-drop crystallization experiments were enabled through specially designed and 3D-printed adaptors. These adaptors accommodate crystal systems presented on 18 and 22 mm round or square cover slides or microbridges, such as those used with VDX (Hampton Research, Aliso Viejo, California, USA) or Linbro (MP Biomedial, Santa Ana, California, USA) plates (Section S4, Supplementary Figs. S3 and S4).

## 3. Validation experiments   

To validate the instrument, we assessed user experience (whether the system was easy to use and improved the mounting process qualitatively) and experimental efficacy (does the sealing solution work and does the system deliver sufficient productivity gains?). The device was deployed to the Diamond Light Source XChem facility (Diamond Light Source, 2020 ▸) early in the development process to generate extensive user feedback during the design phase. This phase was followed by controlled experiments designed to demonstrate that an engineered solution to the mounting problem was superior to the existing manual practices. In all of these experiments the Shifter was used in documenting mode, where timestamps and experimental annotations are automatically generated and exported as a CSV file. These files were retrieved and used to calculate mounting rates and productivity, as measured by the numbers of crystals mounted and X-ray diffraction data sets collected.

### 3.1. Droplet-evaporation control   

#### 3.1.1. Methods   

**Figure 4:**
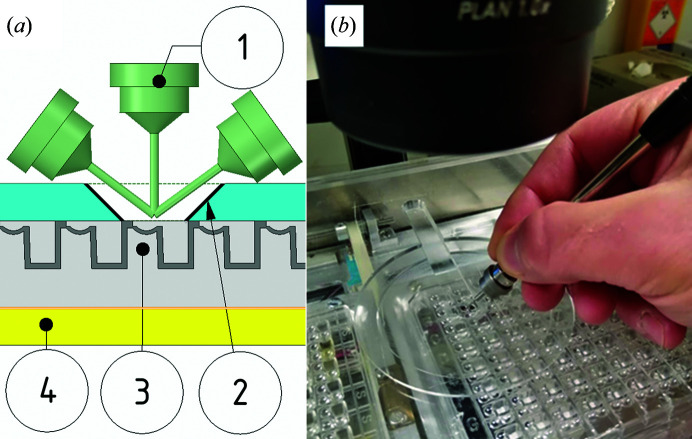
Mounting aperture showing how samples are protected by the lid, seal-free, whilst allowing mounting access to the microplates beneath. (*a*) Cross-section showing a range of mounting angles for the pin (1) through the mounting aperture of the protective lid (2) to the microplate (3) on its carrier (4). (*b*) A pin being used for mounting through the mounting aperture.

Controlling sample evaporation during mounting prevents droplet drying and decreases the dehydration of crystals, which improves unit-cell reproducibility (Farley *et al.*, 2014 ▸). Microplates are usually sealed with an adhesive film during storage, which must then be excised when mounting. The Shifter avoids film cutting by holding microplates, with the storage seal completely removed, against a protective cover. There is a hole in this cover with clearance envelopes around it, such that any part of the microplate can be placed under the hole for mounting whilst all other parts of the microplates remain sealed. The mounting aperture was profiled to provide protection to wells adjacent to the well in use, while allowing a full range of mounting angles (Fig. 4 ▸ *a*).

**Figure 5:**
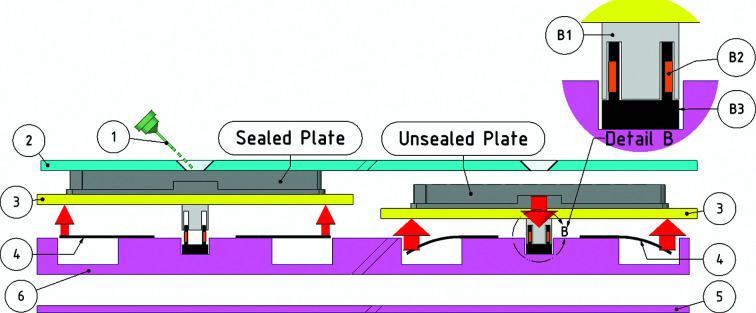
The Shifter mechanism for sealing microplates with the film seal completely removed. (1) Crystal mount pin, (2) acrylic lid, (3) plate carriers, (4) plate support springs, (5) enclosure base, (6) *Y*-axis carriage. Left: the plate shown in its carrier (3) in the sealed position between moves; it is supported by flat springs (4). Right: during a move the plate carrier (3) is pulled down against the force of the supporting springs (4) by electromagnets (Detail B). Detail B: voice coil electromagnets comprising the permanent magnet cap (B1), electromagnetic coil (B2) and coil holder (B3). The red arrows indicate the magnitude and direction of the forces applied to the microplate holders.

When the stage is in motion, the microplates are pulled away from the protective enclosure lid, against the force of supporting springs, by two voice coil electromagnets (MotiCont, Los Angeles, California, USA) on each microplate holder (Fig. 5 ▸). The voice coil motors are de-energized at the end of the move, releasing the microplates and reforming the seal between the microplate and the lid. The two microplate holders can be adjusted separately and set up for different microplate heights and masses.

A prominent issue during crystal harvesting is evaporation of the crystal droplet (Hudaverdyan *et al.*, 2006 ▸). In the absence of a universally used method for the exogenous humidification of samples during mounting, strategies at the SGC have included placing moisture sources around the mounting area or directing the output of an ultrasonic humidifier onto the exposed droplet. These solutions can be exquisitely sensitive to disturbances in air currents within the mounting environment, and in the case of ultrasonic humidifiers can generate an aerosol of water droplets that pools on the work area.

**Figure 6:**
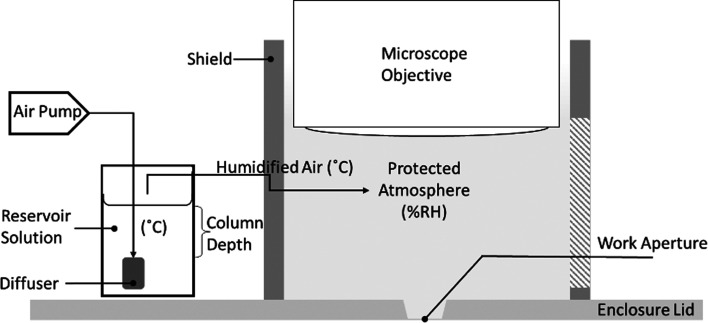
Schematic of the shield and humidifier, made from an aquarium air pump, diffuser (air stone), silicone tubing and a laboratory bottle fitted with a twin-port lid. The pump output (2 × 25 l h^−1^) was connected to a single 19 × 42 mm rod-shaped diffuser.

For this work, a system was developed to control the mounting environment between the microscope objective and the exposed drop using a draught-excluding shield and an improvised humidifier (Fig. 6 ▸).

For a given gas-flow rate, the simplest way to increase the percentage relative humidity (%RH) is to increase the depth of the reservoir column above the diffuser (Al Ashry & Modrykamien, 2014 ▸; for supporting data, see Section S4). Evaporative losses make maintaining the column depth problematic, so it should be fixed to ensure 100%RH across a range of temperatures and then blended with dry air to match the %RH of the crystallization condition (Wheeler *et al.*, 2012 ▸). An approach similar to this was employed by Farley and coworkers in their work to improve the reproducibility of unit-cell parameters using a custom apparatus (Farley *et al.*, 2014 ▸).

To evaluate the amount of droplet evaporation during mounting for these design elements, a simple test was devised wherein 50 nl droplets of 1.5 *M* NaCl were deposited into a microplate and monitored for nucleation as an analogue of droplet evaporation. The time to nucleation was measured for a droplet under the Shifter lid at the mounting aperture with the draught excluder, using a positive control of exposed droplets set on top of the enclosure lid.

#### 3.1.2. Results   

Microplates are loaded into the Shifter without any film seal, relying instead on the mating of the upper microplate surface with the underside of the enclosure lid. We show that the Shifter greatly slows evaporation compared with film cutting and resealing, reducing stresses on the crystals and increasing the time that mounters have to work on the drop before it dries.

Nucleation of aqueous NaCl occurs at 6.1 *M* under ambient conditions. From a starting concentration of 1.5 *M* in 50 nl drops, this represents a loss of 3/4 of the water or approximately 38 nl.

The time to nucleation increased by 36% for lid only (*b*) versus control (*a*) (Table 1 ▸). This is thought to be a result of the partial protection from draughts provided by the mounting aperture and the surrounding depression (Fig. 4 ▸). An additional 10% time to nucleation was realized when a draught-excluding shield was fitted between the mounting aperture and the microscope objective (Fig. 6 ▸). Droplets placed under a wholly covered part of the lid showed a slight initial contraction during equilibration, but no further visible change after 5 h, with no crystallization having occurred.

#### 3.1.3. High positional accuracy with low-cost components   

Electronic sensing and control modules from Phidgets Inc., Calgary, Canada were used to read the encoders and control the stepper motors and *Z*-axis mechanism, as they can be connected directly to a PC via USB without intermediate electronics. The vendor provides software drivers and libraries that support application development in a wide range of operating systems and programming languages. Software applications running on the PC integrate the individually addressable Phidgets modules programmatically. Having a variety of robust modules, which are easy to integrate, costs somewhat more compared with open-source alternatives; however, we found that this convenience greatly accelerated prototyping.

Stepper motors were chosen as they are inexpensive and easily controlled programmatically. They were implemented in a closed-loop arrangement as space constraints do not allow a motor that is large enough relative to the mass of the moving parts, which means that it is not possible to avoid missed motor steps and target overshoot. Because of the considerable slack between the motor shaft and the stage movement, the fixed linear encoders that provide the closed-loop feedback measure the position of the relevant stages, rather than tracking the motor position through something like motor-mounted rotary encoders.

Membrane-potentiometer linear position sensors were used to encode the location of the stage because of their low cost and simplicity of installation. Either resistance or voltage (as a ratio of supply voltage) can be measured across the encoder to determine the position of the stage axis. To relate the encoder values to real-world coordinates, scale tape is applied to the enclosure base along the *X* and *Y* axes. A USB microscope is fixed to the stage so that cross-hairs on the camera image overlay the scales (Section S1, Supplementary Fig. S1*b*). This allows encoder readings to be taken along the scales periodically for the full length of the encoder. Real-world stage coordinates can thus be calculated in real time by converting encoder readings via a polynomial function fitted to the error terms (predicted encoder value − observed value) of the calibration data. Polynomial functions of increasing orders were trialled in order to find the optimal function for decoding the stage position whilst compensating for a lack of positional accuracy caused by the low-cost components used in the stage construction.

We considered implementing continuous, smoothed motions for moving plates, known as ‘tool paths’, where a stage or tool follows every point of a predetermined route between locations of interest. Tool paths would require tightly coordinated multi-axis movements, synchronized at a low level electronically, which is not possible with Phidgets. Moving between points of interest on a microplate does not require defined tool paths; thus, we concluded that the current point-to-point movements are sufficient for crystal harvesting and that additional development and expense was not warranted.

#### 3.1.4. Results   

**Figure 7:**
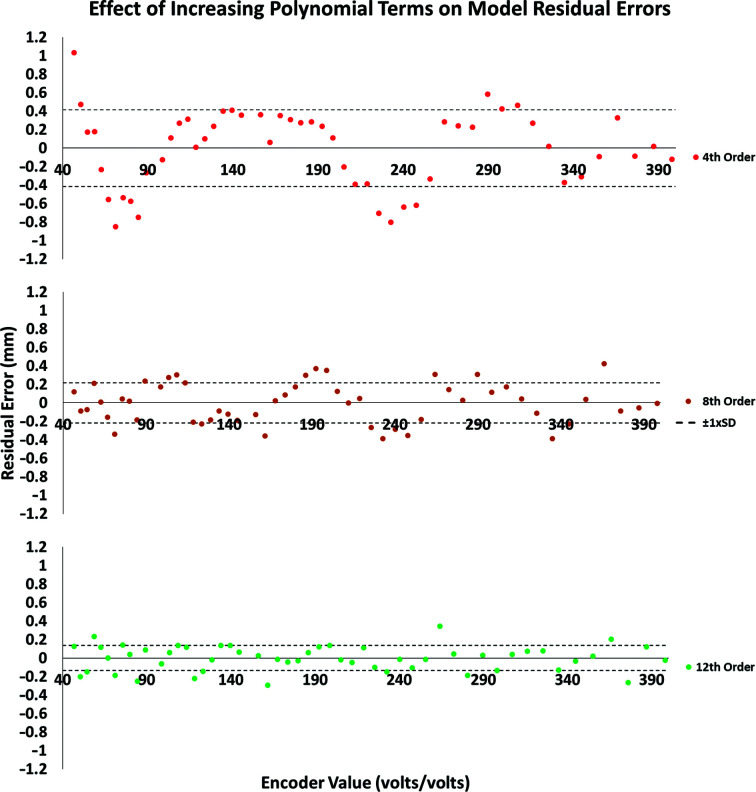
Accuracy of movement can be achieved through calibration. For this typical calibration data set, the fitted polynomial functions all have means for the error residuals that are not significantly different from zero. However, the residuals for lower-order (2–5) and middle-order (6–8) polynomials usually fail the assumption of normality. Higher-order polynomials show no trend over the full length of the stage-position encoder. As the numbers of orders included increases (8–12), the variation of the fitted model to the real-world coordinates becomes acceptable for the current application (4th, SD 0.4 mm; 8th, SD 0.2 mm, 12th, SD 0.1 mm).

By using low-cost linear encoders, the positional resolution of the stage is related not to the step size of the motors (typically 25 µm) but to the resolution of the encoders. Errors in the accuracy of the encoders come primarily from nonlinearity of the sensor (3% according to the manufacturer), which is apparently owing to non-uniform thickness of the sensor. Evaluation of functions to encode values to real-world coordinates found that a 12th-order polynomial function achieved a monotonic relationship between the sensor values and stage position. This function robustly generates well distributed residual errors, with a positional accuracy of 0.1–0.15 mm, over a range of representative calibration data sets. Although this is coarse for a typical *X*–*Y* stage, it is easily compensated for by human aptitude, and thus engineering complexity is kept down, reducing costs (Fig. 7 ▸).

### 3.2. Increasing mounting productivity   

#### 3.2.1. Methods   

A productivity baseline for mounting crystals was established by surveying mounters at the SGC (Structural Genomics Consortium) about practices encountered in the community and expected mounting rates. Self-reported data from the six respondents was used to calculate mounting rates measured in crystals mounted per hour or minutes required per mounted crystal.

Shifter data, saved as CSV files from XChem user sessions for the period September 2015 to January 2016, were aggregated and analysed for patterns of behaviour from academic and industrial users.

A comparison was also made between mounting rates using the manual mounting process (cut and reseal film) and the Shifter-assisted process for a novice mounter (NDW, author 1) and an experienced crystallographer (PC, author 2) to test how mounters of different experience respond to the Shifter (study protein DacA; d-alanyl-d-alanine carboxypeptidase).

Finally, a case study was carried out to explore the burden associated with the training and familiarization of users with the Shifter. In this study a trainee was given brief instruction (∼10 min) on Shifter use, having had no previous crystal-mounting experience. They then mounted unsupervised using a study protein (Pnp2; purine nucleoside phosphorylase II).

#### 3.2.2. Results   

Self-reported data from a survey of six SGC crystallographers suggests a productivity baseline for manual mounting of eight crystals per hour when list generation, data entry and time spent on all other aspects of manual mounting are included (Section S2, Supplementary Tables S1 and S2). Whilst mounting rates will vary depending on the mounter and the protein system in question, this productivity baseline is consistent with the authors’ experience. In the search for a solution to the mounting bottleneck, it is significant that respondents estimate that between a quarter and a half of mounting time is in reality spent on ancillary tasks (Section S2, Supplementary Table S1), and as such this represents a new avenue for process optimization in mounting.

**Figure 8:**
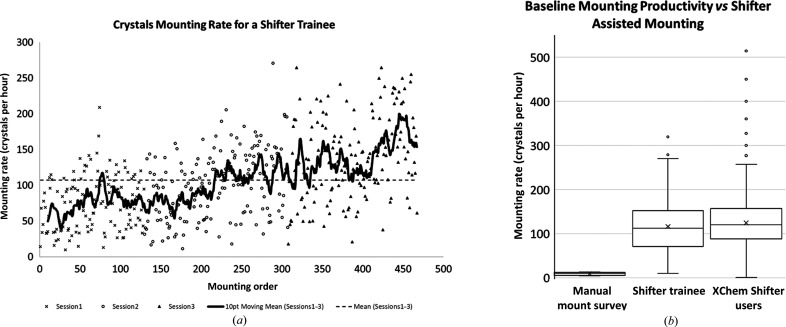
(*a*) A Shifter trainee quickly achieves higher productivity. After 250 crystals, this novice mounter achieved a typical rate of 130 crystals per hour; (seconds per hour divided by the mount duration in seconds). (*b*) A comparison of mounting productivity. The results of the SGC estimated ‘manual mounting survey’ rate (Section S2, Supplementary Table S2), the ‘Shifter trainee’ and data from ‘XChem Shifter users’ (September 2015–January 2016; Section S3, Supplementary Fig. S2). All data are calculated from automatically stored timestamps generated by the Shifter GUI. Mount duration is the difference in time between the requested drop arriving at the mounting aperture and the user requesting to leave it.

XChem users mounted 8271 crystals from at least 17 crystal systems using the Shifter in the first four months of its deployment (September 2015 to January 2016). Experimental outcomes automatically captured by the Shifter GUI show that 86% of mounts were judged to have been a ‘success’ by the user, with a mean mount time of 35 s (103 crystals per hour) and a median of 30 s (120 crystals per hour) (Fig. 8 ▸ *b*). This is in contrast to the productivity baseline estimate of 7.5 min per mounted crystal.

To see whether the Shifter could act as a shortcut to the greater productivity that comes with being an experienced mounter, we compared mount durations between a novice (NDW) using the Shifter and an expert (PC) mounting manually, but found no difference in mount duration. This method was sensitive to a difference between novice and expert when both used the Shifter (*p* < 0.00). We conclude that Shifter-assisted novices can become as productive as manual experts, but that the Shifter increases productivity for all levels of mounter.

In separate case study of a ‘Shifter trainee’, after 10 min of training, mean mounting rates of 75 crystals per hour were seen initially, rising to 140 crystals per hour after 5 h of accumulated mounting time (Fig. 8 ▸ *a*). The starting productivity rate was an order of magnitude faster than the survey baseline and continued to become a lot quicker, at a rate of 22 crystals per hour per each additional 100 crystals mounted (*R* ^2^ = 0.75).

### 3.3. Increased X-ray data retrieved from crystal trials   

#### 3.3.1. Methods   

**Figure 9:**
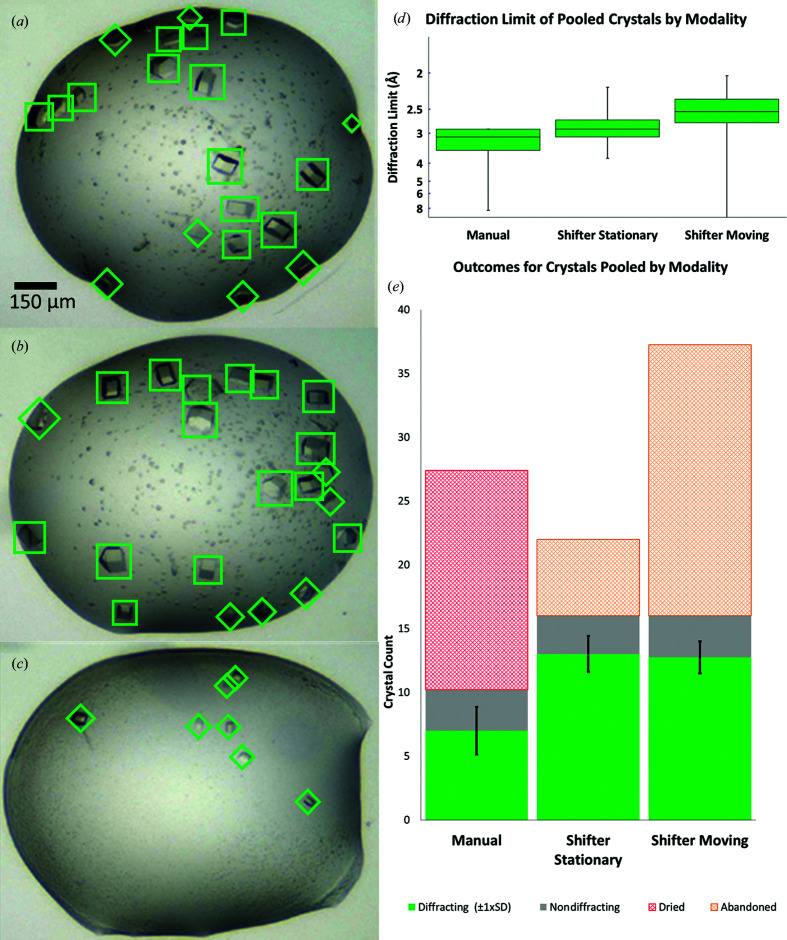
The Shifter enables more crystals (NUDT7A) to be mounted from every drop. (*a*, *b*, *c*) Well drops containing multiple crystals were identified from images based on the number of easily accessible 35–75 µm crystals (green squares). 10–35 µm or inaccessible crystals were counted as second-choice targets, which might be of interest under real conditions (green diamonds). Microcrystals were not counted. The images shown in (*a*), (*b*) and (*c*) relate to experiment 1 in the ‘Shifter Stationary’ series (Fig. 10 ▸, middle panel). (*d*) The X-ray diffraction limits were similarly distributed for crystals mounted from Manual and Shifter experiments. (*e*) Practical considerations limited us to one UniPuck per well (16 crystals). For Manual wells, the drops dried out before reaching this limit (red hatching). For the Shifter wells this quota was met, necessitating that the remaining viable crystals be abandoned (orange hatching).

We measured productivity by the numbers of crystals mounted and X-ray diffraction data sets collected. To test for any effect from using the Shifter, we set up microplates of NUDT7 (Nudix hydrolase 7α; Velupillai *et al.*, 2018 ▸) with conditions known to give an abundance of crystals of 35–75 µm in size. Droplets were imaged and assessed for the presence of such crystals, which are easily accessible to a novice mounter (NDW; Fig. 9 ▸). Second-choice target crystals (10–35 µm in size or those that were poorly accessible) were also documented as they might be a valuable data source in real situations. Microcrystals of <10 µm were not counted, as although they can be mounted using the Shifter, this is not typical for our current workflows. The microplates used have three subwells in each of the 96 well locations; when a well is unsealed, all three subwell drops in that well are exposed. In this experiment, an initial subwell drop was chosen from a suitable well and mounted from for as long as was possible. When all accessible crystals in the initial drop had been mounted, the other drops in the exposed well were used. A cohort of five wells were mounted from using the traditional manual method of cutting and removing the seal (‘Manual’). A second cohort was mounted with the microplate placed in the Shifter, but with the stage stationary between each mounting event (‘Shifter Stationary’). A third group was mounted using the Shifter with a stage move between mounts to simulate a soaking step or similar (‘Shifter Moving’). The collected crystals were then evaluated to determine the X-ray diffraction limit.

#### 3.3.2. Results   

In the experiment to quantify the effect of the Shifter on the numbers of crystals mounted and data sets collected, we saw that for the Manual wells, once the initial drop became unusable all adjacent drops were also unusable. In the Shifter experiment wells the adjacent drops were still viable for mounting, and the diffraction limits for crystals collected from these subwell drops were not significantly different from crystals from the initial drops (Figs. 9 ▸ *a*–9 ▸ *c*; *t*-statistic 2.000, *p* = 0.14).

Mounted crystals yielding a diffraction data set for the Shifter experiments showed a significant improvement in the success rate of mounting over the Manual process (Fig. 9 ▸ *d*; Shifter Stationary, *t*-statistic 1.9, *p* < 0.001; Shifter Moving, *t*-statistic 1.9, *p* < 0.001). No significant difference exists between the two Shifter experiments. When all drops from each cohort were pooled, we also found an improvement in the high-resolution limit for diffraction from the Manual crystals to Shifter Stationary (*t*-statistic 2.48, *p* = 0.018) and from Shifter Stationary to Shifter Moving (*t*-statistic 2.47, *p* = 0.015). This suggests that stage movements provide a small additional improvement in crystal survival on top of the highly significant improvement over the Manual process.

**Figure 10:**
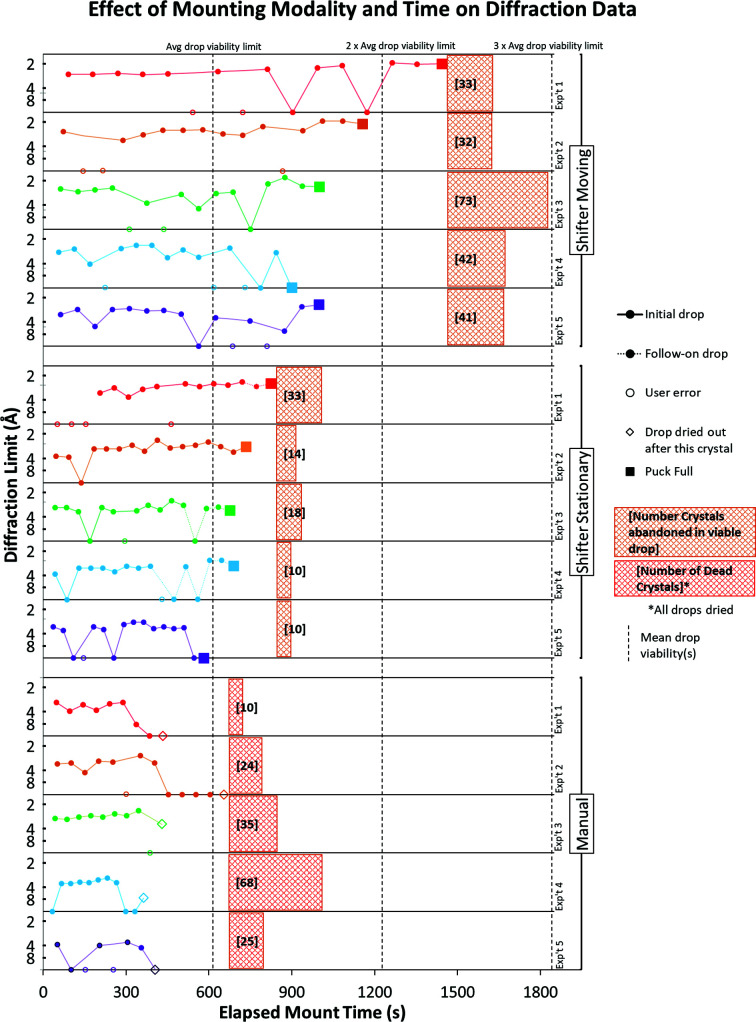
The Shifter enables mounting over a longer time frame. The last crystal mounted from Shifter Stationary initial drops, before they became unusable, has a mean time stamp of 10.2 min. Using mean drop viability as a benchmark, dashed vertical lines indicate expected survival times for successive drops.

In Manual experiments the drops dried with many crystals still present. In Shifter experiments the droplets remained viable (Figs. 9 ▸ *a*–9 ▸ *c*), leading to more crystal mounts and more and higher resolution data sets (Fig. 10 ▸). Although limited to 16 samples per experiment, many of the Shifter drops were still yielding viable crystals over time frames that were long enough to have fully utilized all three drops. The hatched areas include first-choice (35–75 µm) and second-choice (10–35 µm) crystals left behind in viable drops (orange hatching; Shifter trials) or dried drops (red hatching; Manual trials).

It should be emphasized that if a particular crystal system is difficult to mount from, then the Shifter will not in itself alleviate this specific problem (Section S6). Nevertheless, we have observed repeatedly that the improved ergonomics provided by the Shifter appear to facilitate the same improvement for all user-experience levels and degrees of crystallization system mounting difficulty.

## 4. Discussion   

The work presented here not only confirms the major factors that currently make crystal harvesting a bottleneck and experimentally unreliable, but also demonstrate that low-tech approaches can significantly mitigate the problem. In particular, it is the repetitive nature of the overall experimental workflow, with many tedious organizational tasks, that make it error-prone for humans yet eminently suited to automation. What need not be automated is the involvement of human visual acuity, dexterity and sensory feedback, which remain crucial for crystal manipulation but have consistently proven to be exceptionally difficult and expensive to mechanize. Thus, the central design premise of the Shifter was to integrate the strengths of a human operator with an adaptable, motorized hardware platform and a software application, resulting in a semi-automated workflow of crystal harvesting that achieves high productivity despite not being fully automated. This approach also avoids the need for advanced robotics and complex engineering solutions, instead compensating for the inaccuracies that stem from low-cost engineering both by relying on operator adaptability and implementing careful calibration in the software.

Achieving a lost-cost solution was a specific strategic goal, as it is the most realistic way that improvements in experimental efficiency will make a wide impact. This applies both in crystallography laboratories beyond XChem and in fields outside protein crystallography where samples are located in microtitre plates.

The Shifter removes from the operator the need to track physically annotated lists of target drops, manually moving the plate around but also holding it still, and recording experimental data. Operators can thus focus directly on mounting and everything happening within the crystal drop, and are thus far more efficient, and since data are handled automatically, the duration of the entire harvesting workflow is reduced. This division of labour between mounter and Shifter not only significantly narrows the productivity gap in crystallography, but also provides a route to encode experimental best practice for novices while lowering the level of dexterity that they need to master.

It was seen from the manual mounting survey (Section 3.2) that the bottlenecks in mounting come as much from nonmounting tasks such as data administration, microplate movements and seal cutting as they do from crystal handling. The greatest increases in productivity we observed came from adding a clear GUI to the stage motorization, as was shown by the improvements in productivity seen during GUI development, as more of these activities were automated or eliminated. Although GUI optimization was not an initial objective, it later became a key part of the design process (Leikanger *et al.*, 2016 ▸; Kriesi *et al.*, 2016 ▸): the project was ultimately successful because user workflows were integrated and the GUI optimized, enabling single operators to carry out high-throughput experiments that previously required two people. Similarly, it will mainly require adaptions to the software to make the Shifter applicable to experiments outside crystallography, specifically those involving the manual transfer of samples within and between microplates.

The Shifter has reduced the time taken to fill a 16-sample puck to 10 min from 60–80 min. Harvesting rates of 100 crystals per hour can thus be routinely achieved, sometimes reachng as high as ≥240 crystals per hour. The device also alleviates the need for working in a cold room when this is performed solely to extend drop survival during mounting.

The enclosure lid assists in the organization of hand tools and significantly steadies the hand by providing a resting surface for wrists. The method of sealing by lid and the sealing method prevent noticeable evaporation for as long as 30 min, which is the typical duration of a harvesting session. The extended droplet viability and the reduction in sample deterioration allows operators to retrieve many more crystals from a single drop: the Shifter can thus also be applicable to low-throughput settings.

The initial exploratory development was greatly accelerated through exposure to real-world use, specifically in the Diamond-based XChem facility for crystal-based fragment screening (Collins *et al.*, 2017 ▸). This technique intrinsically entails large numbers of mounted crystals, so the mounting bottleneck was a critical impediment to the XChem workflow. These exacting experimental needs of the facility ensured that the Shifter actually achieved the increases in productivity.

In a more general crystallographic setting, improving the harvesting experiment should lead to reduced work upstream in protein purification and crystallization, leading to quicker conclusions to the overall structural biology experiment, and accordingly a reduced per-outcome cost, similar to as has been observed in other fields (Adams, 2008 ▸; Pareek *et al.*, 2011 ▸; Wetterstrand, 2016 ▸).

The Shifter has the potential to enable many more crystallographic experiments than those reported here. To date it has only been used at room temperature, but the enclosure lends itself to cooling to low temperatures, thus accommodating crystal systems that must be maintained at 4°C, requiring the operator to be in a cold room. Initial tests successfully maintained the temperature inside the enclosure below 0°C and above 90%RH for extended periods without condensation internally or externally; the remaining challenge is in integrating a suitable air-cooling and delivery mechanism.

### 4.1. Observations on developing hardware as a bench scientist   

As barriers to prototype engineering continue to fall, the community of scientists who are used to improvising laboratory apparatus is presented with great opportunities for innovation. Nevertheless, the ultimate impact of such projects requires a clear and realistic understanding of what it takes to drive them to completion.

Thanks not least to the evolution of movements such as Open Source and Maker, along with ‘freemium’ pricing models from software providers, end users have access to tools to create bespoke apparatus at low cost and without formal design training (Lian *et al.*, 2009 ▸; Pearce, 2013 ▸). Starting at least as far back as the introduction of programmable integrated circuits in the 1970s (Augarten, 1974 ▸), and continuing with popular microcontroller-powered electronics modules, end users are similarly able to generate sophisticated sensing and control systems without in-depth electronics or software training. The large user communities of these modules have ensured economies of scale that have allowed them to make their way even into low-volume commercial products (Cooke, 2017 ▸; Fryer, 2014 ▸).

This project tapped heavily into these evolutions, but nevertheless was subject to a common feature of hardware projects: if the project goal is to deliver a stable, reproducible and independently operable product, the hardware development will remain a protracted and thus difficult process. This contrasts strongly with the software aspects of the project, and indeed equivalent software initiatives overall, where design and release cycles should be rapid (Beck *et al.*, 2001 ▸).

What is common to hardware and software is that designs must be thoroughly exposed to real-world use in order to uncover design limitations and highlight where resources need to be invested or what can be omitted. It is only such an iterative process of ‘hardening’, whether time-consuming or not, that will reveal where user needs really lie and whether the audience will ultimately see value in the solution.

In our view, the reproducibility crisis (Pashler & Wagenmakers, 2012 ▸; Staddon, 2017 ▸) extends to the many hardware projects reported in the literature: articles tend to focus on the aspects of a development that are most interesting to the intended users of a technological development, but without including adequate technical detail to assess and reproduce a proposed design. Links to 3D print files or code repositories are not sufficient to enable the reproduction of a result in hardware development, especially if this requires as much further development as it would to design something from scratch. In this respect, there is an unmet need either to publish complete assemblies in a portable CAD (computer-aided design) format or to provide more detailed manufacturing and assembly instructions. Such sharing of complete designs, even physical copies, is commonly seen in, for example, the sharing of reagents and plasmids between laboratories.

On the other hand, this means that such homegrown designs, in order to be widely effective, will ultimately still require commercialization, where the first major task will be to close the large distance between the published design and a marketable product. The corollary is that even designs with unencumbered intellectual property can be successfully commercialized, since the competitive advantage resides in the expertise and the amount of work required to close this gap. All it needs is for most users to find it easier to buy a device than to try and reproduce one.

As in all areas of design, in MX the technological road to improving quantity and quality has no shortage of proposed solutions that have failed to achieve community penetration (Hammack, 2014 ▸). The hurdle is that existing experimental solutions have proven themselves to be useful in spite of their weaknesses, and new solutions will thus struggle to be adopted and deliver impact if they are not user-ready. They must also integrate with existing practices, or else be wholly independent of external dependencies (Weissenberger, 2013 ▸), at least until their performance is persuasive to the bulk of the user base (Tellis, 2006 ▸). By not requiring a major retooling of commonly used labware or changes to the larger experimental workflow, the Shifter appears to accommodate these constraints to uptake.

## 5. Conclusions   

Despite the tremendous success of X-ray cryo-crystallography in recent decades, crystal harvesting remains a manual process in almost all laboratories. The microscope stage described here transforms crystal mounting from a rate-limiting manual process to a high-throughput semi-automated process. The visual acuity and fine motor skills of humans are combined with targeted hardware and software automation to transform the speed and robustness of crystal mounting, automatically capturing experimental annotations. The Shifter was engineered to have a simplified design that can be commercialized at low cost and therefore be adopted widely beyond fragment screening and protein crystallography.

Since 2015, a series of Shifter devices have been deployed as part of the XChem fragment-screening facility at Diamond Light Source, where they have since facilitated the mounting of over 120 000 crystals, including most recently 3000 for the COVID Moonshot program (Chodera *et al.*, 2020 ▸), leading to 71 fragment binders for the SARS-CoV-2 main protease (MPro; Douangamath *et al.*, 2020 ▸).

## Supplementary Material

Supplementary Information including Supplementary Figures and Tables. DOI: 10.1107/S2059798320014114/tz5103sup1.pdf

## Figures and Tables

**Table 1:** The Shifter dramatically increases the nucleation time of salt crystals from 1.5 *M* NaCl solution The data show a reduction in droplet evaporation for plates loaded into the Shifter (*b*, *c*, *d*) compared with unsealed plates (*a*).

Test location	Mean time to nucleation (s)	Improvement versus (*a*) (s)	Improvement versus (*a*) (%)
(*a*) Positive control (open)	277		
(*b*) Mounting aperture only	377	100	36
(*c*) Mounting aperture + draught excluder	404	127	46
(*d*) Covered deck position	>18000		
