# Supplementary material for: The low-cost Shifter microscope stage transforms the speed and robustness of protein crystal harvesting
Source: Acta Crystallogr D Struct Biol. 2021 Jan 1;77(Pt 1):62–74. doi: 10.1107/S2059798320014114 (PMC7787106; doi:10.1107/S2059798320014114)
Supplement: Supplementary file 1 [file d-77-00062-sup1.pdf]

STRUCTURAL BIOLOGY

Volume 76 (2020)

Supporting information for article:

The low-cost Shifter microscope stage transforms the speed and robustness of protein crystal harvesting

Nathan David Wright, Patrick Collins, Lizbé Koekemoer, Tobias Krojer, Romain Talon, Elliot Nelson, Mingda Ye, Radosław Nowak, Joseph Newman, Jia Tsing Ng, Nick Mitrovich, Helton Wiggers and Frank von Delft

S1. Encoder Connectivity and Calibration

Encoder counts are related to real-world coordinated by comparing the encoder output to fixed scale markers.

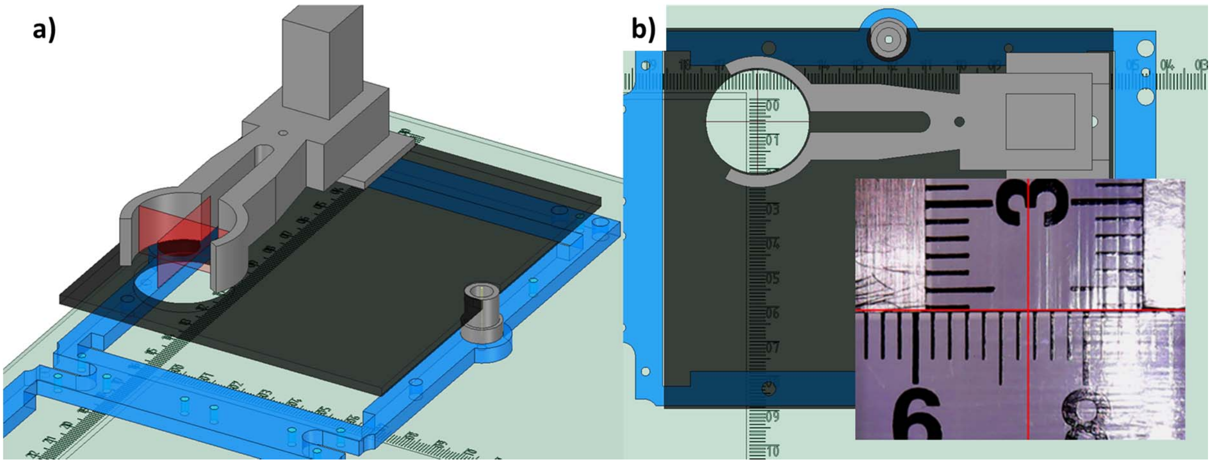

Figure S1 Calibrating the encoder: **a)** A USB microscope is clipped into a support (grey), which is fixed to a base (black). This assembly is mounted on the Y Carriage (blue). **b)** Cross-hairs (red) on the camera image overlay the X and Y scales on the enclosure base (green).

S2. Survey of SGC Mounters on Time Spent Mounting

Six crystallographer responded to a self-response survey of time use and mounting practices as encountered in the community.

Survey Question:

Q: "How long does it usually take you to manually mount a puck of crystals (16 pins)?"

*A: Preparation/Pre-mounting (preparing mounting lists, etc.)*______________________________

A: Mounting (all related processes; cutting seals, finding wells, making notes etc.) ____________

*A: Post-mounting (transcribing notes, registering in database, etc.)*____________

Table S1 Time used per phase of mounting process (minutes)

User	Preparation	Mounting	Post-mounting	Total	Time 'off task' (%)
1	10	60	10	80	25
2	45	120	60	225	47
3	12	50	15	77	35
4	20	40	15	75	47
5	30	60	30	120	50
6	20	150	20	190	21

Manual mounting is slow and distracted. Self-reported data show long mount times (M: 8min, SD: 4min) of 7.5 crystals/h, with 38% of time devoted to non-mounting tasks.

S3. XChem User Data: September 2015 and January 2016

Crystal mounting outcomes captured via GUI button press during user operation. ‘Mounted_x’ indicates a successful mount followed by a descriptor of the drop appearance; ‘Mounted_Clear’ is the typical mode for optimised systems.

Table S2 Time used per phase of mounting process (minutes)

Outcome Mode	Number of Mounts	(%)
Fail_Evaporated	66	1
Fail_Melted	933	11
Fail_Unpickable	150	2
Mounted_BadDispense	135	2
Mounted_Clear	6112	74
Mounted_Crystalline	320	4
Mounted_Precipitate	555	7
All Failures	1149	14
All Successes	7122	86
Total	8271	

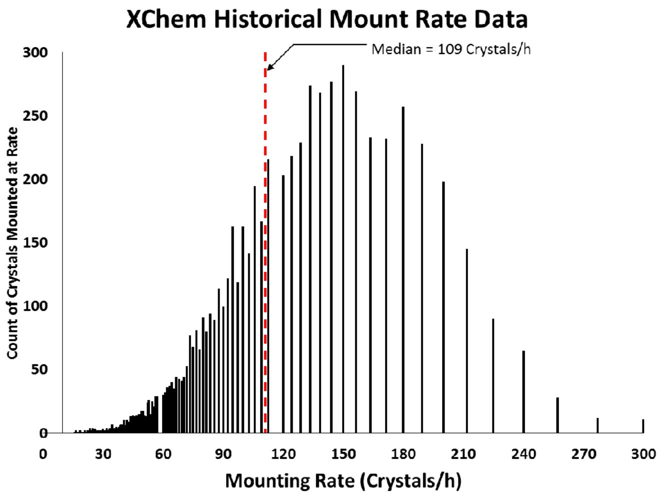

Figure S2 Mount rates of users from the same dataset as Supplementary Table S2. The mount duration for each mounted crystal was recorded in whole seconds, and converted to an hourly rate with the following formula:

$$Mounting\ Rate = \frac{Crystals}{hour} = \frac{3600(\frac{s}{hr})}{Crystal\ Mount\ Duration\ (s)}$$

S4. Droplet humidification techniques

Evaluation of bubble-column humidifier configurations, against the practice of using a domestic ultrasonic humidifier.

Table S3

Humidification Type	Reservoir Solution	Reservoir Temp. (°C)	Configuration (Column depth or power setting)	Protected Atmosphere Humidity (%RH)	Humidified Air Temp. (°C)
Bubble	Water	4	70ml	42	21
Bubble	Water	83	70ml	96	30
Bubble	5M NaCl	20	70ml	65	20
Bubble	1.5M NaCl	20	70ml	70	20
Bubble	Water	20	70ml	74	20
Bubble	Water	20	140ml	98	20
Ultrasonic*	Water	20	Min. power	97	20
Ultrasonic*	Water	20	60% power	98	20

*ultrasonic humidifier at the work area.

S5. Large droplet format capacity

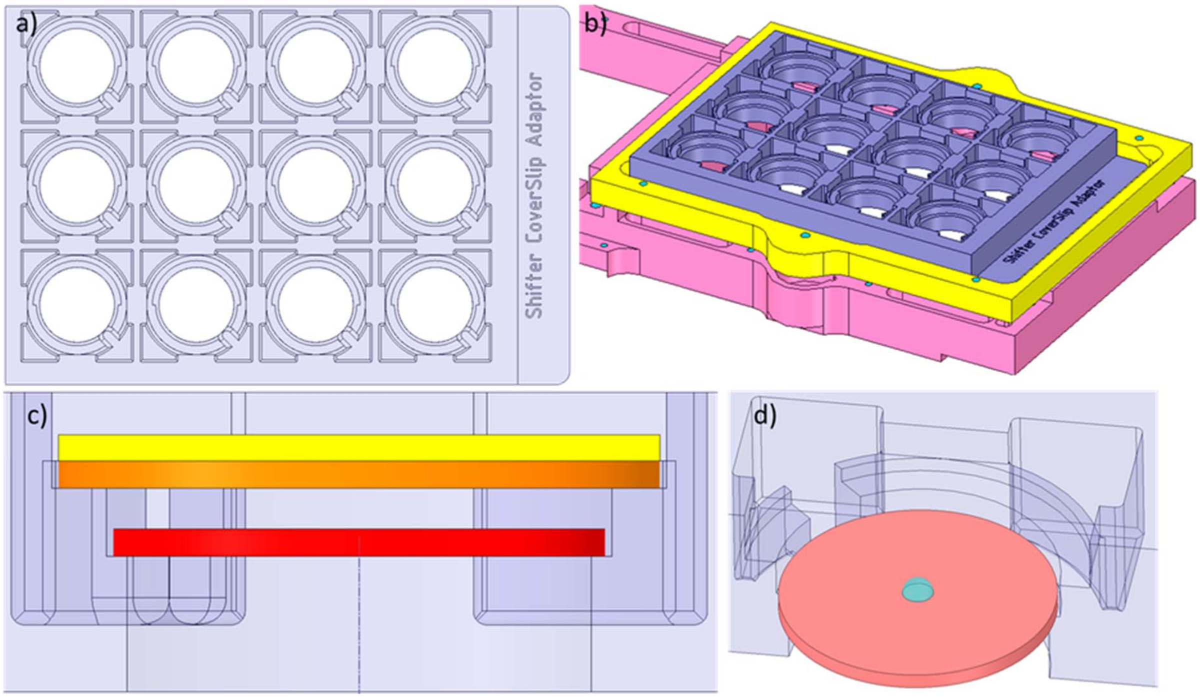

Figure S3 Cover slip adaptor for inverted hanging-droplet experiments **a)** plan view, **b)** trimetric view, **c)** Cross-section and **d)** cutaway, showing position of 18mm (red) and 22mm (orange) round, and 22mm square (yellow) coverslips.

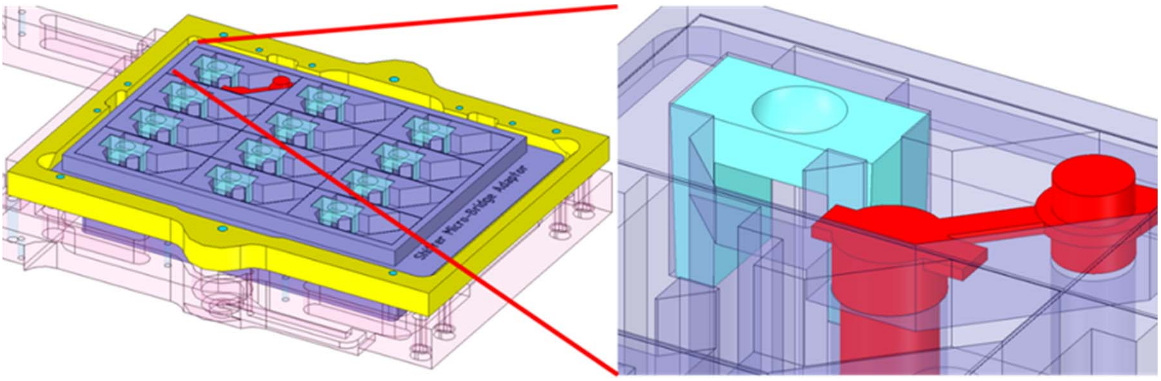

Figure S4 Micro-Bridge (light blue) adaptor with removable 0.2ml PCR tube reservoir (red)

S6. Mounting from difficult drops remains somewhat difficult

An experienced mounter (RT, author 3) harvested from a selection of crystal systems judged to be representative of differing mounting difficulty: BRD1A (Bromodomain-containing 1a) (easy mounting), DacA (moderately easy), and JMJD1BA (Lycine Demethylase) (difficult mounting). Median mounting times were 25, 20 and 29 seconds per crystal respectively (124-180 crystals/h) (Supplementary Fig. S5). Whilst there is no trend between 'mounting ease' and absolute speed of mounting in this experiment, mounting does seem to become less skewed as the difficulty of retrieving crystals increases. Mount times of 13-15 seconds per crystal seem to represent the practical

limit of the mounting process, not because of the speed of the Shifter (move times 1-3 seconds are typical), but because of mounting, freezing and storing the crystal, and preparing for the next mount. Whilst the Shifter cannot 'cure' the effect of a difficult system on mounting speed, metrics captured by the system now allow for routine analysis of such trends.

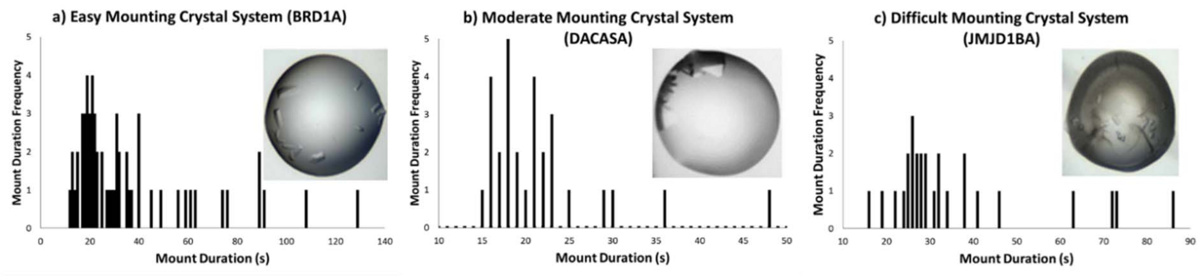

Figure S5 Crystal mount time distributions: a) BRD1A n=59, median 25s; b) DacA n=29, median 20s; c) JMJD1BA n=27, median 29s
